# Non-Communicable Diseases: The Invisible Epidemic

**DOI:** 10.3390/jcm11195939

**Published:** 2022-10-08

**Authors:** Daniele Piovani, Georgios K. Nikolopoulos, Stefanos Bonovas

**Affiliations:** 1 Department of Biomedical Sciences, Humanitas University, Pieve Emanuele, 20090 Milan, Italy; 2 IRCCS Humanitas Research Hospital, Rozzano, 20089 Milan, Italy; 3 Medical School, University of Cyprus, Nicosia 1678, Cyprus

Historically, communicable diseases, such as HIV/AIDS, viral hepatitis, malaria, poliomyelitis, tuberculosis, influenza and, more recently, the coronavirus disease 2019, have been at the center of global health concerns and initiatives, as they are transmitted from one person to another with a variety of ways, easily spread across national borders, and threaten the lives of millions of people all over the globe. Nonetheless, it is the “invisible epidemic” of non-communicable diseases (NCDs) that represents the world’s leading cause of death [1]. 

NCDs, also known as chronic diseases, are characterized by non-contagious nature, multiple risk factors, a long latency period, a prolonged temporal course, functional impairment or disability, and incurability (i.e., a complete cure is rarely achieved) [2]. They are responsible for over 41 million deaths each year, a figure that is equivalent to 74% of all deaths globally, whereas many more millions of people are living with NCDs and experience a reduced quality of life. The dominant types of NCDs are four: cardiovascular diseases (including heart disease and stroke; 17.9 million deaths per year), cancer (9.3 million deaths per year), chronic respiratory diseases (including chronic obstructive pulmonary disease and bronchial asthma; 4.1 million deaths per year), and diabetes mellitus (2.0 million deaths per year) [3,4]. 

The list of NCDs that impose heavy morbidity and mortality toll also includes neurological and mental health diseases (e.g., Alzheimer’s disease and other dementias, Parkinson’s disease, depressive disorders, substance use disorders, and schizophrenia), glaucoma and hearing loss, digestive diseases (e.g., peptic ulcer, liver cirrhosis, and inflammatory bowel diseases), musculoskeletal diseases (e.g., rheumatoid arthritis, osteoarthritis, osteoporosis, gout, back pain, and trauma), chronic kidney disease, autoimmune conditions, and others [5]. 

Importantly, NCDs increasingly affect poor countries, deprived societies, and the poorest people within all nations, as the “westernization” of lifestyle gradually takes its toll in low- and middle-income countries, population ages, and commercial pressures for unhealthy diets and harmful habits continue to exist [6]. Indeed, of all deaths from NCDs (i.e., the more than 41 million deaths occurring each year, globally), one-quarter occur in high-income countries while approximately three-quarters occur in the low- and middle-income countries where most of the world’s population lives. It is important to note that 17 million people die from NCDs each year before reaching 70 years of age, with the majority (86%) of these premature deaths occurring in low- and middle-income countries [3,4]. 

The tragedy (and opportunity) is the fact that most NCD morbidity and mortality could be prevented or delayed, and millions of people could live longer, healthier and happier lives [3]. Several factors can increase the risk of developing NCDs: they can be classified as modifiable or non-modifiable factors (e.g., age, gender, genetic factors, race, and ethnicity). The modifiable factors can also be classified into: (i) metabolic/biological (e.g., excess weight/obesity, hyperglycemia, hyperlipidemia, and raised blood pressure); (ii) behavioral factors (e.g., unhealthy diet, tobacco use, physical inactivity, and harmful use of alcohol or other substances); and (iii) societal factors, which involve complex combinations of interacting socioeconomic (e.g., poverty, low public spending on health, limited access to health services) and environmental parameters (e.g., climate change, sunlight, and air pollution) [7]. 

Importantly, it is estimated that most NCD deaths can be prevented by eliminating the most important modifiable factors [8], particularly the four behavioral risk factors for NCDs, i.e., tobacco use [9], unhealthy diets [10], misuse of alcohol [11] and physical inactivity [12], which in turn lead to the metabolic/biological risk factors described above. The currently available scientific evidence provides a strong basis to justify taking immediate action against the NCD epidemic; beyond the necessary medical care for those already affected, the adoption of NCD prevention strategies is considered to be the most cost-effective, affordable and sustainable course of action to cope with the chronic diseases’ burden worldwide. 

NCDs will be the predominant global public health challenge of the 21st century [1]. Global prevention and control strategies should aim to decrease the incidence of NCDs, delay disease onset and disability, alleviate disease severity, and improve the health-related quality and duration of the individuals’ lives [13]. Health promotion activities (raising awareness, encouraging healthy lifestyles and addressing modifiable risk factors), early detection programs (i.e., screening populations at high risk for NCDs), and initiatives aiming to manage chronic diseases and related complications (so that their progress is slowed or stopped), can play a key role in controlling the “invisible epidemic” of NCDs, on the national and the global level.
